# Evaluation of promoters and ribosome binding sites for biotechnological applications in the unicellular cyanobacterium *Synechocystis* sp. PCC 6803

**DOI:** 10.1038/srep36640

**Published:** 2016-11-18

**Authors:** Elias Englund, Feiyan Liang, Pia Lindberg

**Affiliations:** 1 Department of Chemistry - Ångström, Uppsala University, Box 523, SE-751 20 Uppsala, Sweden

## Abstract

For effective metabolic engineering, a toolbox of genetic components that enables predictable control of gene expression is needed. Here we present a systematic study of promoters and ribosome binding sites in the unicellular cyanobacterium *Synechocystis* sp. PCC 6803. A set of metal ion inducible promoters from *Synechocystis* were compared to commonly used constitutive promoters, by measuring fluorescence of a reporter protein in a standardized setting to allow for accurate comparisons of promoter activity. The most versatile and useful promoter was found to be P*nrsB*, which from a relatively silent expression could be induced almost 40-fold, nearly up to the activity of the strong *psbA2* promoter. By varying the concentrations of the two metal ion inducers Ni^2+^ and Co^2+^, expression from the promoter was highly tunable, results that were reproduced with P*nrsB* driving ethanol production. The activities of several ribosomal binding sites were also measured, and tested in parallel in *Synechocystis* and *Escherichia coli*. The results of the study add useful information to the *Synechocystis* genetic toolbox for biotechnological applications.

Cyanobacteria have gained increasing attention as sustainable converters of CO_2_ and H_2_O into valuable products using solar energy^1^. However, engineering of cyanobacteria for production of such compounds often requires the introduction and expression of multiple genes in a well-controlled manner. This, in turn, requires well characterized and tightly regulated promoters. Having well controlled promoters is also important in cases where an engineered pathway is producing a toxic intermediate or product, which may lead to genetic instability and loss of expression if the pathway is constitutively expressed.

There are many different promoters to choose from for use in the unicellular cyanobacterium *Synechocystis* sp. PCC 6803 (from here on referred to as *Synechocystis*). Strong native promoters such as the *psbA2* and *rbcL* promoters, the recently described “super strong” P*cpc560*^2^ or the J23-series of promoters^3^ can all be useful when an “always on” constitutive expression of genes is possible or desired. However, for applications such as induced cell lysis, use of the CRISPR-Cas9 system or stable integration and expression of genes with a high metabolic burden, inducible promoters are required. Currently, there is a shortage of useful, well characterized inducible promoters for *Synechocystis*. Many inducible systems commonly used in *Escherichia coli* do not function as well in *Synechocystis* due to differences in cellular properties or growth requirements. Examples include the case of LacI not completely releasing the repressing of P*trc* or P*tac* promoters, preventing high induction levels^4^,^5^ and problems when using the light sensitive anhydrotetracycline to induce expression from the Tet-promoter during phototrophic growth^6^. The green light inducible promoter P*cpcG2* system described in Abe *et al*.^7^ could be useful for specialized growth conditions, but will be inherently difficult to work with during growth under normal white light.

An alternative set of regulated promoters are metal ion inducible promoters that have already been used for several applications^8^,^9^,^10^,^11^,^12^,^13^. In *Synechocystis*, a set of genes responsible for Ni^2+^, Co^2+^, and Zn^2+^ tolerance are all grouped together in a gene cluster^14^. The *nrsBACD* operon encodes a set of transporters which provide protection against Ni^2+^, and is induced by both Ni^2+^ and Co^2+^. Directly upstream of *nrsBACD* is the *nrsRS* operon, encoding a two-component signal transduction system where NrsS senses Ni^2+^ ions and phosphorylates NrsR which activates the transcription of *nrsBACD* and *nrsRS*^15^. *nrsD* is also transcribed from a promoter between *nrsC* and *nrsD*, which is repressed by InrS in the absence of Ni^2+ ^16.

The gene responsible for Co^2+^ resistance is *coaT*, encoding a cobalt exporting ATPase, the expression of which is induced by both Co^2+^ and Zn^2+^ ions^14^. In the presence of Co^2+^, it is transcriptionally activated by CoaR, encoded upstream of *coaT*^17^. In the absence of Co^2+^, CoaR acts as a repressor of *coaT*. Zn^2+^ resistance is mediated by ZiaA which is repressed by ZiaR in the absence of Zn^2+^. *ziaR* is transcribed upstream of *ziaA* together with *sll0793*, which codes for a membrane bound protein likely affiliated with Zn^2+^ transport^18^.

A frequent biotechnological application for cyanobacteria is metabolic engineering for production of a desired compound. In those utilizations, the metabolic burden placed on the cell, in the amount of resources needed to make the product and the heterologous enzymes, as well as the depletion of substrate for other pathways, can lead to severe growth reduction and genetic instability^19^,^20^. Examples include the production of mannitol where mutants commonly reverted to the wt phenotype^21^, the production of isobutanol where one of the enzymes commonly reduced its function^22^ and the production of lactic acid where an NADH:NADPH transhydrogenase could not be kept stable in the wt^23^. Partly because of the issue with genetic instability, metabolic studies have utilized inducible promoters. In cyanobacterial strains which have a functional induction of P*trc* and P*lac* such as *Synechococcus elongatus* PCC 7942, those are commonly used^24^. For *Synechocystis* production, P*nirA* have been used for production of carotenoids^25^, P*nrsB* for manoyl oxide production^12^ and P*coaT*, P*petE*, and a version of the Lac promoter were used for ethylene production^13^.

For biotechnological applications, promoters should be easy to use in genetic constructs, have a low leakiness, a strong induction and allow for fine-tuning the levels between those extremes. The aim of this work was to identify and characterize practical promoters and a set of ribosomal binding sites (RBS) for biotechnological purposes in *Synechocystis*, and to do so in a systematic way, using standardized conditions and genetic setting to allow for careful analysis and comparisons. We did not aim to investigate the functional elements or initiation dynamics of each promoter. Earlier studies have examined the transcriptional activation of some of the metal inducible promoters^26^,^27^. Here we expand on that work by providing systematic, useful data quantitatively comparing these promoters to each other and to some commonly used promoters, with fluorescent protein levels as well as formation of a model product, ethanol, as reporter.

## Results and Discussion

### Choice and cloning of promoters

For this study, we chose to characterize the promoters P*nrsB*, P*nrsD*, P*nrsS*, P*coaT* and P*ziaA*, all of which drive the expression of nickel, cobalt and zinc metal efflux pumps in the same gene cluster, and the Cu^2+^ inducible plastocyanin promoter P*petE* which has been frequently used for expression in *Synechocystis*^28^. For comparison, we also included a selected set of other native promoters from *Synechocystis*: the strong promoter driving expression of the D1 subunit of Photosystem II; P*psbA2*, in three versions differing by sequence length (P*psbA2*S, P*psbA2*M, and P*psbA2*L, see Supplementary Fig. 1 for sequence details), the photosystem I subunit Ia promoter P*psaA*^29^, the RubisCO large subunit promoter P*rbcL*1A^4^, and RNase P subunit B promoter P*rnpB*. All the promoters were PCR amplified from *Synechocystis* genomic DNA and cloned into the self-replicating vector pPMQAK1^4^ in front of the strong ribosomal binding site RBS*^28^, followed by the gene encoding Enhanced yellow fluorescent protein (EYFP)^30^, and the terminator BBa_0015^31^. We used an RBS calculator (https://salislab.net/software/forward)^32^ to predict if the presence of the native RBS on the promoters sequences would start translation prematurely, instead of at the synthetic RBS we added directly upstream of EYFP. For the promoter sequences where the native RBS were predicted to still be functional, 9 base pairs were truncated from the 3′ end of those sequences. For P*rnpB*, the native promoter of an RNA gene which does not have an RBS, we used the entire promoter sequence up to the start of the gene, and an RBS was added. The complete sequence of all promoters used in this study can be found in Supplementary Table 1.

### Determination of fluorescent reporter levels from promoter constructs

Promoter constructs were transferred into *Synechocystis* by conjugation and an empty vector control was included. The concentrations of metal ions used to induce the promoters were chosen based on a balance between having high enough concentration to get a high induction and low enough to not stress the cells. For *Synechocystis*, the half growth-inhibitory concentration (IC_50_) of Ni^2+^ was previously reported to be 27 μM, 8 μM for Co^2+^, between 8 and16 μM for Zn^2+^, and 2 μM for Cu^2+ ^27. Our initial designation of standard metal inducer concentration was 5 μM for Ni^2+^ and 4 μM for Zn^2+^, as per the recommendations of Blasi *et al*.^27^. The Co^2+^ concentration used was 6 μM, and 0.5 μM for Cu^2+^, as in Guerrero *et al*.^13^. The BG11 medium used to cultivate *Synechocystis* contains trace amounts of some of the metal ions used to induce expression^33^. There is no Ni^2+^ added in BG11, but it does contains 0.17 μM Co^2+^, 0.77 μM Zn^2+^, and 0.32 μM Cu^2+^, which potentially could induce the expression of some of the promoters. However, the purpose of our study was to investigate how these promoters can be used in a biotechnological application, where an absence of certain metals could affect the function of proteins responsible for vital processes in the cell, and a strict requirement for keeping the growth medium free from some metals would be impractical and likely not economically feasible. Therefore, all experiments were done using complete BG11, including the trace metals, except in certain control experiments as indicated below.

**Figure 1:**
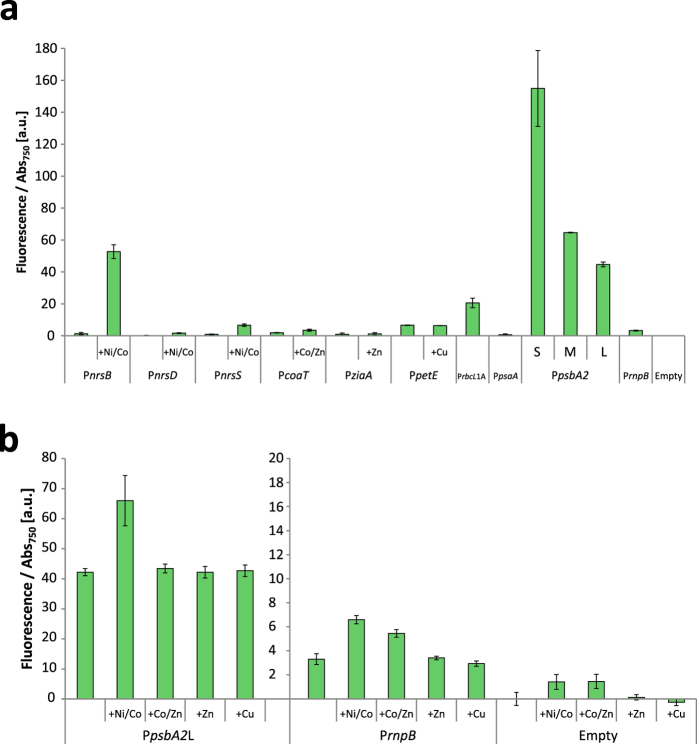
Promoter activities. (**a**) Activity of promoters in *Synechocystis*, as measured by EYFP fluorescence. (**b**) Effect of metal induction on fluorescence for control strains. Two y-axis scales are used to better visualize low expressed samples. Promoter activities were measured as EYFP fluorescence per Abs_750_ two days after induction by addition of metal ions. The fluorescence of the un-induced empty vector control strain was subtracted from each sample. Metal ion concentrations used for induction were 5 μM Ni^2+^, 6 μM Co^2+^, 4 μM Zn^2+^ and 0.5 μM Cu^2+^. Error bars represent SD (*n* = 4).

*Synechocystis* cells carrying the respective promoter constructs were used to inoculate fresh cultures, which were grown for two days before induction of expression by addition of the various metal ions. The cells were cultivated for an additional two days, and then fluorescence from expressed EYFP was determined (Fig. 1a).

The Ni^2+^ and Co^2+^ induced promoter PnrsB showed a low leakiness and a high induction rate, going from half of the levels of the reference promoter P*rnpB* to a 39-fold increase of expression levels. The other two promoters from the nickel tolerance system, P*nrsD* and P*nrsS*, were induced fourteen and seven times, respectively, but had a lower maximal expression than P*nrsB*. The Co^2+^ induced promoter P*coaT* showed a low expression and low induction by its inducer metals Co^2+^ and Zn^2+^, while the Zn^2+^ dependent promoter P*ziaA* had very weak activity both in the presence and absence of added Zn^2+^. The *petE* promoter showed no change in expression upon addition of Cu^2+^, likely due to it already being fully induced by the 0.32 μM Cu^2+^ that is present in BG11.

For the strong photosystem II promoter P*psbA2*, the length of native sequence included in the promoter had a large effect on resulting expression levels, with the short version having more than three times higher expression than the long one. A difference between the promoters is that P*psbA2*S lacks the beginning of an antisense RNA which has a role in stabilizing the *psbA2* mRNA^34^. However, due to the stabilization effect of the antisense RNA, one would expect higher expression with the full sequence intact, and that would also require a promoter sequence within the EYFP gene to promote the transcription of the antisense RNA, which to our knowledge there is not. Another study identified repressor binding sites in P*psbA2*, deletion of which resulted in increased expression of the promoter^35^. All of those binding sites reported in that study are still present on the three versions of P*psbA2* used in this study, but our results suggest a possibility of additional regulatory sites even further upstream.

Although the P*psbA2* is one of the strongest native promoters, expressing one of the most abundant gene transcript in the cell^36^, and being commonly used for heterologous chemical production^37^,^38^,^39^, it is still weaker than the synthetic P*trc* promoter. That is important to consider when choosing a promoter since the limitation for production is commonly enzyme availability, something that has been seen for several products, including lactic acid^40^,^41^, ethanol^42^, β phellandrene^43^, and ethylene^44^. By normalizing the P*psbA2* expression observed here to P*rnpB* and comparing with another study where P*trc* and P*rnpB* were measured^6^, the inferred differences in expression are 1.8, 4.2 and 6.1 times weaker for P*psbA2S*, P*psbA2M* and P*psbA2L* respectively compared to P*trc*1O.

Curiously, even though the photosystem I promoter P*psaA* has been widely used for transgenic expression in *Synechocystis*, we could only detect a minimal expression from this promoter in our construct. We used the short A4 sequence version of P*psaA* from Muramatsu and Hihara^29^. A possible cause for the lack of expression is that the combination of that promoter with the EYFP and RBS sequences used created an unfavorable 5′UTR, preventing efficient expression^45^.

### Metal-ion effect on fluorescence signal in control strains

As a control experiment, we wanted to investigate whether there is an effect of addition of metals on accumulation of fluorescent protein, even when expressed from the native constitutive promoters. For these experiments, we chose P*psbA2*L and P*rnpB* as reference promoters. P*psbA2*L was picked for its similar strength to the induced levels of P*nrsB*, and P*rnpB* because it is considered to be constitutive under standard cultivation conditions^46^. The addition of metals to the reference strains increased fluorescence for some of the metal combinations, up to twice the levels of the non-metal induced cultures, but much lower than the effect on fluorescence from the P*nrsB* construct induced with Ni^2+^ (Fig. 1b). To our knowledge, neither P*psbA2* nor P*rnpB* are directly metal inducible, and even the empty vector strain that did not contain the EYFP gene slightly increased in fluorescence, indicating that the effect was unspecific.

It is known that the fluorescent signal will increase if the cells grow slower due to slower dilution of EYFP proteins, which is the main mechanism of EYFP removal^47^. This effect could be somewhat diminished by addition of a protein degradation tag on EYFP, targeting it for continuous degradation^4^. However, this option was not explored in this paper, since our aim was primarily to study the usefulness of the investigated promoters for expression of proteins, not the dynamics of expression. Instead, due to this unspecific increase in fluorescence, we decided that for all further comparisons we would treat control promoters and metal induced promoters with the same induction conditions, so as to not over-estimate the specific effect of induction.

### Expression at different inducer concentrations

**Figure 2:**
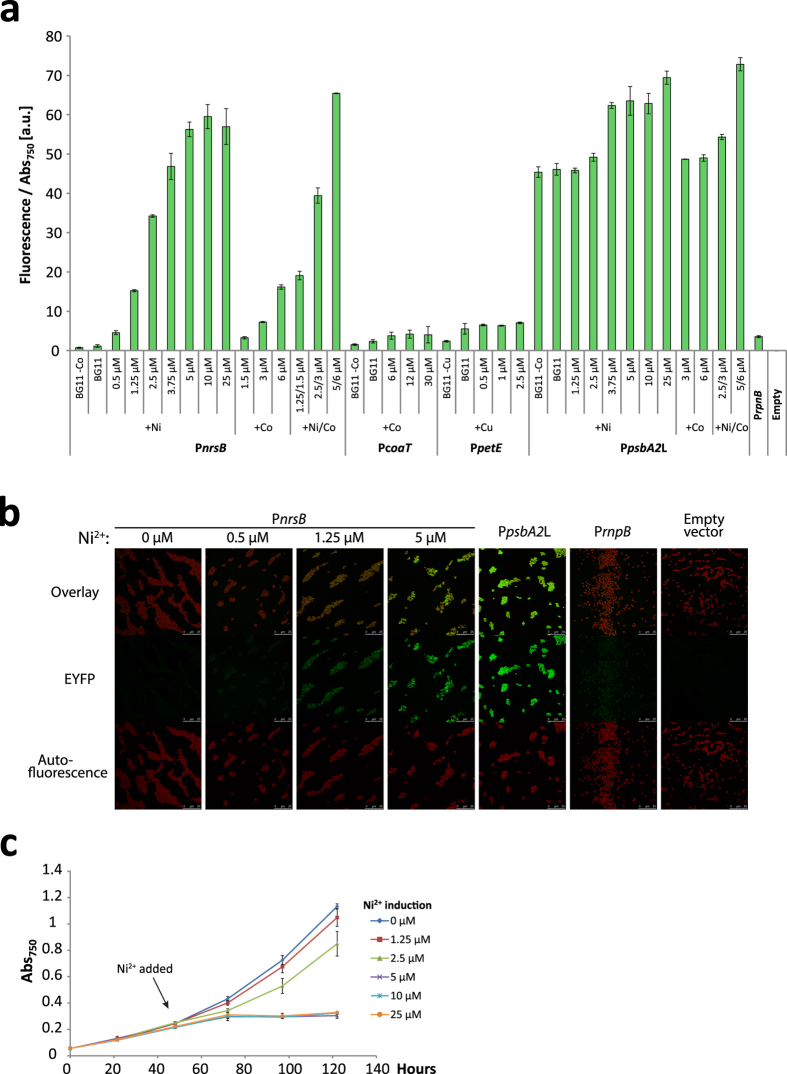
Metal ion induction effects on promoter activity and cell growth. (**a**) Promoter activities measured as EYFP fluorescence per Abs_750_ two days after induction. The fluorescence of the empty vector control strain was subtracted from each sample. P*rnpB* and the empty vector strain were grown in BG11. Error bars represent SD (*n* = 4). (**b**) Fluorescent microscopy image of cells expressing EYFP driven by P*nrsB* at different Ni^2+^ induction strengths, P*psbA2*L, P*rnpB* or the empty vector. Bottom panels: chlorophyll autofluorescence, middle panels: EYFP fluorescence, top panel: overlay of EYFP and chlorophyll signals. (**c**) Growth of wild type *Synechocystis* with different amounts of Ni^2+^. Cells were grown at 12 μmol photons m^−2^ s^−1^ and Ni^2+^ was added after 48 h. Error bars represent SD (*n* = 3).

To test the range of induction of P*nrsB*, P*coaT* and P*petE*, and to see how much each inducer contributed to the promoter activity, the cells were subjected to a range of concentrations of their inducer metal ions. In this experiment, the BG11 medium used was modified to not include the inducing metal ion(s). Starting from Co^2+^-free BG11, there was an increase of the fluorescence in cultures with the P*nrsB*-driven EYFP construct with increasing concentrations of Ni^2+^ which plateaued at 5 μM (Fig. 2a). Addition of both inducers, Ni^2+^ and Co^2+^, gave a response similar to when only Ni^2+^ was used, and induction with only Co^2+^ gave 3.5 times lower expression than with 5 μM Ni^2+^. Both for P*coaT* and P*petE*, using Co^2+^ or Cu^2+^ higher than the initial level of induction did not lead to higher expression, but removal of the inducer metals from BG11 cut the expression in half, compared to complete BG11.

To determine if the lower expression of P*nrsB* at reduced Ni^2+^ induction concentrations was due to a lower expression level in every cell in the culture or a binary reduction where the culture becomes a mixture of on and off cells, laser scanning confocal microscopy was used. Cells with the P*nrsB* promoter at induction concentrations 0 μM, 0.5 μM, 1.25 μM and 5 μM showed a clear and continuous increase in EYFP fluorescence, spread out across all cells (Fig. 2b). This indicates that at lower inducer concentrations, less transcription occurs at the cell level, and not only on the population level.

As described above, we based our initial selection of inducer concentration on literature data. To see at which concentration nickel had detrimental effects on growth in our cultures, wild type *Synechocystis* cells were grown for 48 h and then subjected to different amounts of Ni^2+^. Already at 5 μM, there was a large reduction of growth while at 2.5 μM, the effect on growth was less severe (Fig. 2c). Due to the relatively high induction of P*nrsB* (Fig. 2a) and low toxicity of Ni^2+^ at 2.5 μM, we decided to use that as the standard concentration for the following experiments.

### Measurement of promoter activity over time

**Figure 3:**
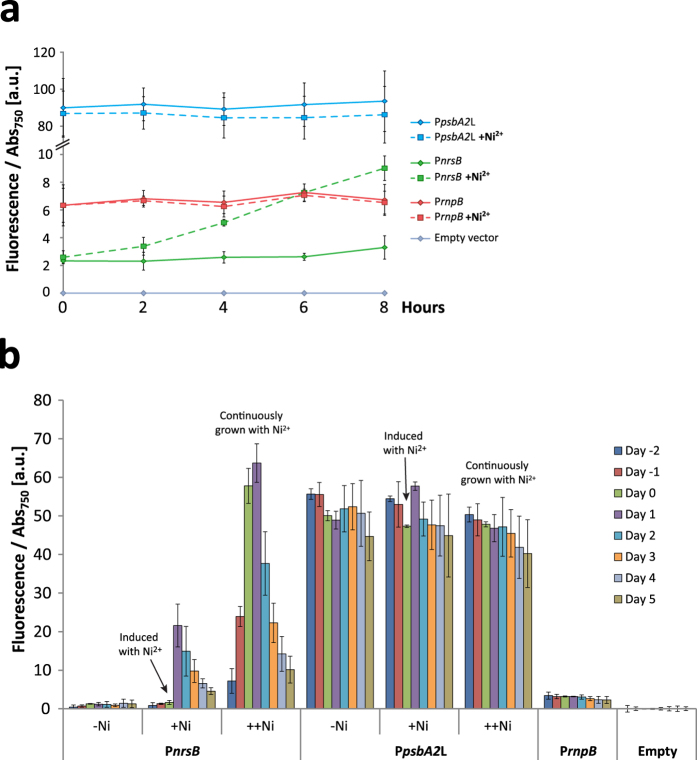
Promoter driven EYFP signal change over time. Promoter activity measured as EYFP fluorescence per Abs_750_. (**a**) All strains were induced with 2.5 μM at 0 hours and measured every second hour. (**b**) The fluorescence of strains with and without induction was measured for eight days. The P*nrsB* and P*psbA2*L strains were either grown in BG11 (−Ni), with addition of 2.5 μM Ni^2+^ at day 0 (+Ni) or grown continuously with 2.5 μM Ni^2+^ (++Ni). P*rnpB* and the empty vector strain were grown in BG11 without Ni^2+^. The fluorescence of the empty vector control strain was subtracted from each sample for each day or hour. Error bars represent SD (*n* = 4).

Unlike most inducible promoter systems, use of metal inducible promoters has an additional complication in that the inducer molecule is being actively pumped out of the cells. Therefore, the concentration of metal ions inside the cells might change as more efflux pumps are induced, even though the metal ion concentration in the medium remains constant. To determine how the induction of P*nrsB* changes over time, fluorescence was measured over the course of a day and the course of a week. When the response of P*nrsB* to Ni^2+^ was measured over eight hours, the induction had led to a detectable increased expression already after 2 hours (Fig. 3a). To see how expression varied over a longer time period, the EYFP accumulation across eight days of growth was assayed in strains growing without induction, with induction on the second day, and with Ni^2+^ continuously present in the medium (Fig. 3b). In the P*nrsB* strain, the highest amount of fluorescence was observed when reaching the early stationary phase (~OD_750_ 1.3, “Day 1” in Fig. 3b) after which the fluorescence went rapidly down, something not seen in the P*psbA2* strain. This peak in fluorescence in the P*nrsB* strain could possibly be caused by a lowered concentration of Ni^2+^ transporters in each cell due to cell division during the rapid growth occurring the days before the peak, increasing the intracellular Ni^2+^ concentration. It was also seen that continuous growth with nickel gave a higher maximal induction than when induced after 2 days.

One way to prevent the export of the inducer would be to integrate the genes to be expressed in the *nrsBACD* operon itself, thereby have them be expressed by the native P*nrsB* while at the same time knocking out the nickel export system. An *nrsBACD* knock out strain would have a reduced tolerance to Ni^2+ ^14, but might also allow for induction at lower metal concentrations. However, this option was not explored within the scope of this paper.

### Ethanol production with P*nrsB*

**Figure 4:**
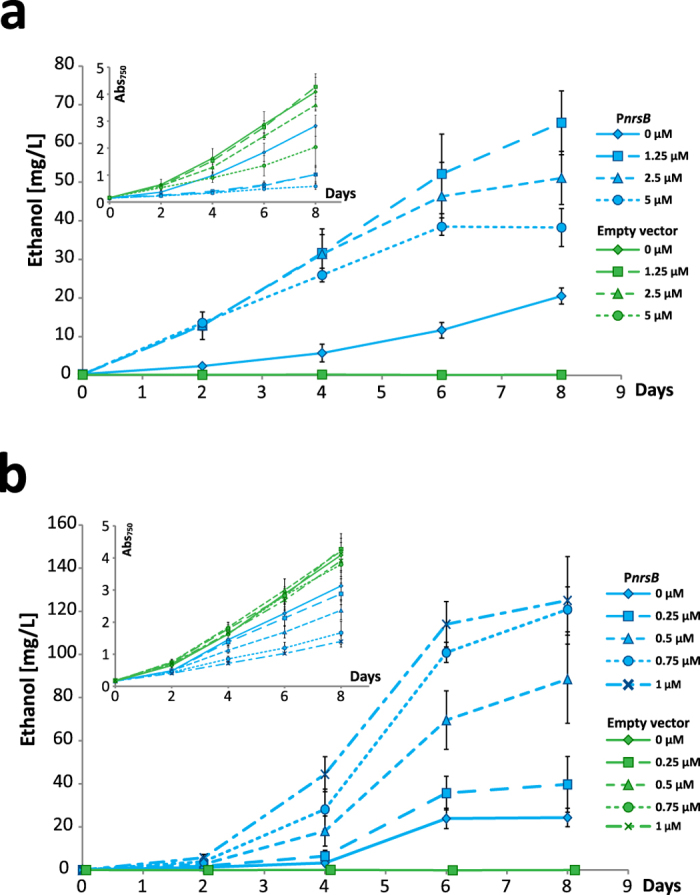
Ethanol production and growth with varying amount of Ni^2+^ induction. A strain with P*nrsB* driven expression of *pdc* and *slr1192* and the empty vector strain were induced with a range of Ni^2+^ concentrations, from (**a**) 0–5 μM Ni^2+^ or (**b**) 0–1 μM. Error bars represent SD (*n* = 4).

To evaluate the potential usefulness and capacity of the investigated promoters for actual biotechnology applications, and to get a measure of the total promoter expression output across several days, we made a new construct with P*nrsB* driving the expression of genes for ethanol production^48^. Expression patterns from reporter molecules and metabolic products can differ, hence the need to measure P*nrsB* activity using a product based reporter system^49^. Since ethanol readily diffuses across the cell membrane and accumulates in the media, we can use ethanol accumulation as a reporter to compare the effect of different promoters for the expression. Pyruvate decarboxylase (*pdc*) from *Zymomonas mobilis* and the native *Synechocystis* alcohol dehydrogenase (*slr1192*)^42^ were cloned in a new P*nrsB*-driven construct, which was introduced into *Synechocystis*. The resulting strain was grown with different concentrations of Ni^2+^, and ethanol accumulation and growth were measured. Over the course of 8 days, the highest ethanol accumulation in the medium reached 65 mg/L (Fig. 4a).

The cultures grown with 1.25 μM Ni^2+^ had the highest ethanol production, which was somewhat surprising, since the EYFP data suggested that higher induction than that would increase expression further, and in a previous study, it was shown that ethanol production could be increased several times upon gene duplication of *slr1192* and *pdc,* effectively increasing the expression levels of the enzymes^42^. However, a clear decrease in growth could be observed in the ethanol producing strain upon addition of Ni^2+^, an effect not seen in the empty vector control. When measuring the ethanol at lower induction levels, from 0–1 μM Ni^2+^, the production increased with increasing amounts of Ni^2+^ while the growth rate decreased (Fig. 4b). It may be that at higher concentrations of the inducer, the combined stresses of Ni^2+^ exposure and ethanol production counteract any increase in product formation which could result from enhanced protein expression levels per cell. At lower levels of induction, the effects of Ni^2+^ are not as severe, with higher yield as a result.

Attempts to make an ethanol producing strain of *Synechocystis* where the expression was driven by P*psbA2*, in order to compare it with the P*nrsB* driven ethanol production, were unsuccessful. When tested in *E. coli*, the P*psbA2*-driven construct was leading to ethanol production, but in *Synechocystis,* the ethanol producing capability with this construct was rapidly lost (data not shown). We were unable to get a stable ethanol producing strain for any of the three P*psbA2* variants, in agreement with a previous study^50^, while the P*nrsB* strain lost its production after too long exposure to Ni^2+^. This effect may likely be attributed to genetic instability of the ethanol production construct: in experiments where expression levels were higher, a larger proportion of cells in the culture lost their ability to generate ethanol, due to selective pressure on the cells. This has been reported previously as a problem for continuous ethanol production^51^.

The genetic instability of ethanol production highlights the importance of inducible promoters for genetically stable production strains. For complex multi-step pathway products, P*nrsB* could serve as a gatekeeper to prevent genetic instability, controlling expression of the first enzymes in the pathway while a strong constitutive promoter expresses the rest, thereby only completing the full pathway when Ni^2+^ is added.

### Effect of light on P*nrsB* activity

**Figure 5:**
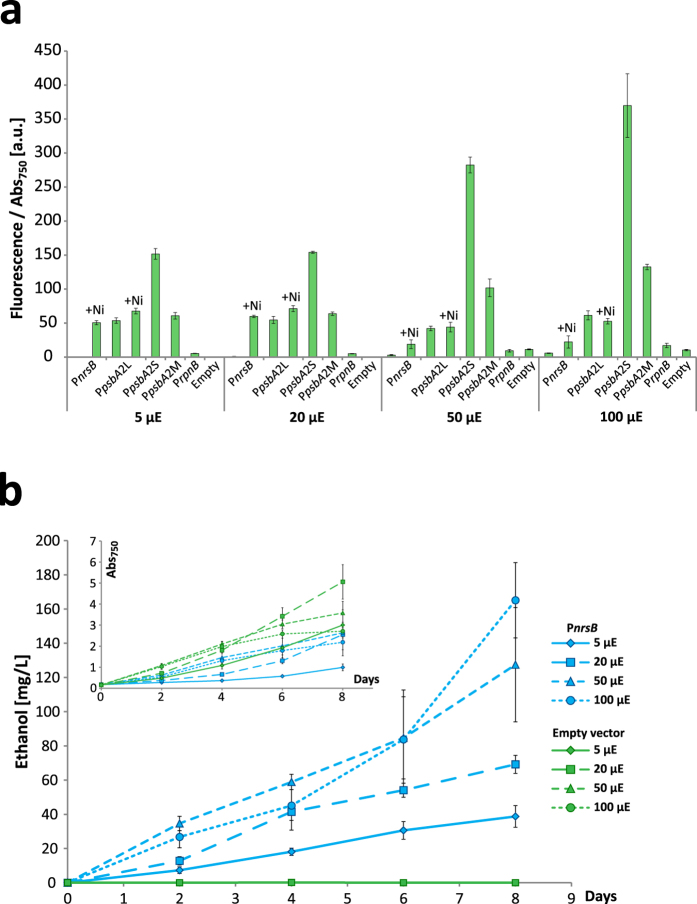
Effect of light intensity on P*nrsB* activity. (**a**) Promoter activity measured as EYFP fluorescence per Abs_750_. Cells were grown at different light intensities and fluorescence was measured two days after induction with 2.5 μM Ni^2+^. The fluorescence of the empty vector control strain at 5 μE was subtracted from each sample. (**b)** A strain with P*nrsB* driven expression of *pdc* and *slr1192* and the empty vector strain were grown at different light intensities, and ethanol and growth was measured. μE = μmol photons m^−2^ s^−1^. Error bars represent SD (*n* = 4).

In a previous study where we used P*nrsB* for the production of the high value compound manoyl oxide, we observed a decrease in production at high light, something we speculated could be due to reduced function of P*nrsB* at higher light^12^. In order to investigate this effect, we performed experiments to determine the influence of light intensity on EYFP expression as well as ethanol production. For P*nrsB*-driven EYFP expression, increasing the light from 20 μE to 50 μE did reduce the induced fluorescence with more than half, with similar reduction at 100 μE (Fig. 5a). The short and medium length sequence of P*psbA2* increased EYFP expression as the light increased, consistent with literature^52^. For the longer version of the P*psbA2* promoter, expression did not increase with higher light intensity, for reasons as of yet unknown. When P*nrsB* was driving the expression of ethanol producing genes, induced with 2.5 μM Ni^2+^, cultures growing at 50 and 100 μE accumulated more ethanol after eight days than cultures grown at 20 μE, even if normalized per biomass (Fig. 5b). This inconsistence with the fluorescence data may be due to increased substrate availability for ethanol production under higher light conditions, leading to more product being formed even if the enzyme expression levels are lower than at lower light.

### Promoter activity in *E. coli*

**Figure 6:**
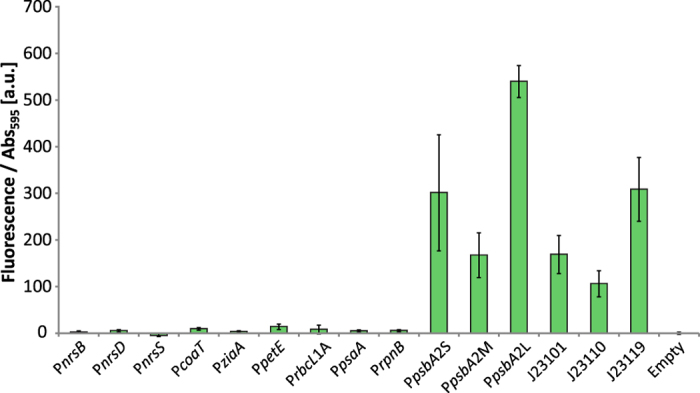
Promoter activities in *E. coli*. Promoter activities measured as fluorescence per Abs_595_. The fluorescence of the empty vector control strain was subtracted from each sample. Error bars represent SD (*n* = 6).

For cloning purposes, it can be beneficial to have the promoter turned off in the cloning host, thus preventing selection pressure to mutate detrimental genes. Therefore, we investigated the *Synechocsytis* promoters tested in this paper for activity in *E. coli*. The promoters J23101, J23110 and J23119 from the Registry of standard biological parts^31^ were used as references for the strength of expression. None of the *Synechocystis* promoters had any discernable activity except P*psbA2* (Fig. 6). The transcriptional machinery of *Synechocystis* has several differences compared to the one in *E. coli*, e.g. a split β´ subunit in the RNA polymerase and a different sigma factor composition^53^, explaining the large differences in expression pattern between the two strains. Of the P*psbA2* strains, the long version had the strongest expression in *E. coli* and the medium length the lowest, a third of the long one. In contrast, expression form P*psbA2* in *Synechocystis* was strongest with the short version while the long version had the weakest expression (Fig. 1a).

### Characterization of ribosome binding sites

**Figure 7:**
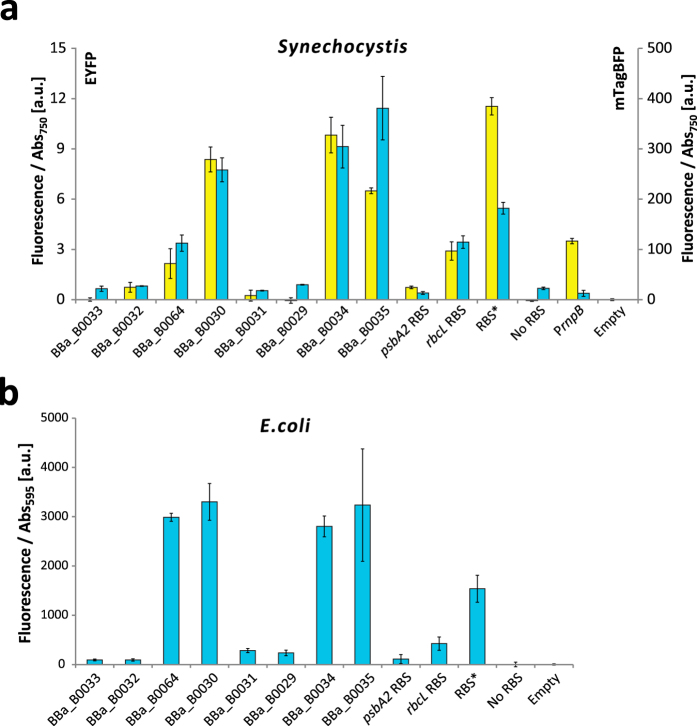
Comparative strengths of ribosomal binding sites. (**a**) Activity of RBSs in *Synechocystis,* measured using EYFP (yellow bars) and mTagBFP (blue bars). The eight RBSs from the BioBrick library are sorted left to right with increasing strength based on values from the literature^31^. All EYFP constructs were expressed by P*petE* and mTagBFP construct by P*psbA2*S except the “P*rnpB*” strain which were expressing EYFP and mTagBFP using P*rnpB* and RBS*. (**b**) Activity of RBSs in *E. coli* using mTagBFP fluorescence. RBS activity was measured by fluorescence per Abs_750_ for *Synechocystis* or Abs_595_ for *E. coli* and the fluorescence of the empty vector control strain was subtracted from each sample. Error bars represent SD (*n* = 4 for *Synechocystis, n* = 6 for *E. coli*).

Other than promoters, the choice of ribosomal binding site can have a large impact on product formation in metabolic engineering studies^44^,^49^. Also, having a library of different strengths of RBSs available can be useful for constructing operons with variable strength of expression of each gene. Therefore, we selected 11 RBS sequences to characterize, eight from the BioBrick Registry of standard biological parts^31^, the two native RBSs upstream of the *psbA2* and *rbcL* genes, and the synthetic RBS*^28^, and compared their expression in *Synechocystis* (see Supplementary Table 2 for sequence details). Because the activity of RBSs can vary highly depending on sequence context^45^, each RBS was measured using two constructs designed to have high sequence dissimilarities in the upstream and downstream sequences from the RBS, one expressing EYFP driven by P*petE,* and the other mTagBFP driven by P*psbA2S*^54^. The eight RBSs from the BioBrick registry had a large variation in expression strength and showed a similar relative activity for both fluorescent markers, indicating that the genetic context did not influence expression in major ways for the two sequences used to test the RBSs in this study (Fig. 7a), although it should be noted that the pattern might not hold for other sequences.The exceptions were BBa_B0035 and RBS*, which both changed in relative strengths, depending on if the RBSs where measured in the EYFP constructs or the mTagBFP constructs. Both BBa_B0029 and BBa_B0031 had weak expression even though they have been reported to have levels comparable to BBa_B0030 in *E. coli*^31^.

All mTagBFP expressing RBS-constructs were also tested in *E. coli*, to see whether RBSs have comparable relative strengths in both organisms. The results showed that the relative strengths of the RBSs to each other were similar when expressed in *E. coli* as in *Synechocystis* (Fig. 7b), consistent with the fact that the *Synechocystis* and *E. coli* ribosomes have the same core anti-Shine-Dalgarno sequence (5′-CCUCC-3′)^55^. This is in sharp contrast to the differences in promoter function, where nine out of ten *Synechocystis* promoters tested in this study were not functional in *E. coli*. This experimentally verified RBS study may prove useful for balancing gene expressions in operons, especially since predicted RBS strengths may not correlate well with the actual strengths^56^.

### Conclusions

In this report, we investigated the activity of a set of native promoters and a library of RBSs in *Synechocystis*, and provide systematic, comparative data on their respective output levels and tuneability. Of the tested promoters, we found that the nickel inducible *nrsB* promoter was the most versatile with a strong, well-regulated expression that can be fine-tuned by varying the concentration of the inducer. We also demonstrated that these characteristics can be utilized for expression of a production pathway, by using P*nrsB* for inducible ethanol formation. Drawbacks in using P*nrsB* include a reduced expression at higher light, an efflux of the inducer which might lead to a variable intracellular concentration, and the toxicity of Ni^2+^ at higher concentrations. However, despite those shortcomings, we believe it is a viable choice for many applications that require inducible expression.

It is important to note that promoter and RBS activity can vary based on genetic context^45^, and thus, depending on the specific genes expressed, the expression strength may be different. Nevertheless, the characterization performed in this study provides useful information on the relative strength of the promoters and RBSs, and enhances our understanding of the induction pattern of the promoters we have examined here.

## Methods

### Vector construction

The promoter sequences from Supplementary Table 1 were amplified by PCR using *Synechocystis* genomic DNA as template with primers flanked by EcoRI and SpeI sites. Using 3A assembly^57^, promoter parts were ligated together with a BioBrick containing RBS*, EYFP and a terminator (BBa_B0015) and then ligated into the broad-host-range shuttle vector pPMQAK1^4^. For PpsbA2M and PpsbA2S, the RBS was included on the primers and thus, no BioBrick scar was created between promoter and RBS. For the ethanol construct, *pdc* was amplified from *Zymomonas mobilis, slr1192* and P*nrsB* from *Synechocystis*, and the parts ligated into pPMQAK1 using 3A assembly. Correctly assembled vectors were verified by sequencing. The vectors were transferred to *Synechocystis* by triparental mating as described previously^58^, using the pRL443 conjugative plasmid. Isolation of correct transformants was verified by PCR amplification of extracted DNA, to confirm presence of the desired constructs.

For the RBS constructs, P*petE* or P*psbA2* were combined with the RBSs, EYFP or mTagBFP respectively, and terminator BBa_B0015 using 3A assembly into pPMQAK1. The “No RBS” constructs was made without RBS, directly linking promoter to the fluorescent protein gene. The EYFP constructs contained the sequence TCTAGA between the RBS and EYFP while the mTagBFP constructs contained that sequence between P*psbA2* and RBS and TACTAG between RBS and mTagBFP. The vectors were conjugated into *Synechocystis* in the same way as for the promoter constructs.

### Cultivation conditions

*Escherichia coli* strain DH5α (Invitrogen) was used for subcloning and cells were grown in LB supplemented with 50 μg mL^−1^ kanamycin. *Synechocystis* PCC 6803 cultures were grown at 30 °C and 20 μmol photons m^−2^ s^−1^ except when otherwise noted, in BG11 medium with addition of 10 μg mL^−1^ kanamycin in 6-well plates containing 4–6 mL of media. For the experiment where fluorescence was measured over eight days, cultures were grown in 25 ml BG11 in 100 ml Erlenmeyer-flasks.

### Fluorescence measurements

Fresh *Synechocystis* cells were seeded to an Abs_750_ of 0.15 and grown for two days. Solutions of metal salts (NiCl_2_ × 6 H_2_O (Merck), ZnSO_4_ × 7 H_2_O (Merck), Co(NO_3_)_2_ × 6 H_2_O (Merck), or CuSO_4_ × 5 H_2_O (Riedel-de Haën)) were then added to the cultures to obtain the appropriate concentrations of metal ions. After another two days, fluorescence and absorbance was measured with a Plate Chameleon V Microplate Reader (Hidex) using Microtest 96-well Optilux Black Assay Plates (BD Falcon) at 485 nm excitation and 535 nm emission for EYFP and 390 nm excitation and 460 nm emission for mTagBFP measurements. Optical density at 750 nm for *Synechocystis* and 595 nm for *E. coli* was used as a measure of the number of cells in the cultures. For each promoter or RBS strain, cells was grown in duplicates and measured with three technical replicates, and each experiment was repeated twice. Standard deviations were calculated from the four biological replicates. For data analysis, the background was subtracted from absorbance and fluorescence measurements using a blank BG11 sample. Fluorescence was then divided by the optical density to get a value representing average fluorescence per cell. The fluorescence per cell value of cells containing the empty vector was subtracted from all samples.

For activity tests in *E. coli*, cultures were grown overnight in LB, then 2 μl was added to 198 μl of M9 media^59^ in a 96-well plate. Cells were grown for 6 hours in triplicates and then fluorescence was measured in the same way as described above.

### Ethanol measurements

Cells were seeded to Abs_750_ 0.15 in 25 ml BG11 with 50 μg mL^−1^ kanamycin and 2.5 μM Ni^2+^ except when otherwise noted. Samples were taken every other day and Abs_750_ and ethanol accumulation was measured. Cultures were grown with four replicates for eight days at 20 μmol photons m^−2^ s^−1^ except when otherwise noted. For measuring ethanol, samples from cultures were pelleted and 1 μl of the supernatant was injected in a Clarus 580 Perkin Elmer FID gas chromatograph (GC) with a packed column (1.8 m × 2 mm i.d., Cat No. N9305013-ZW5531, Perkin Elmer). Injection was done through a packed injector, the carrier gas was N_2_ at 20 ml min^−1^. The GC program was: 130 °C for 5.5 min, then ramp to 230 °C at 45 °C min^−1^ and held for 5 min. The area of the ethanol peak at 4.96 min was converted into mg/L by using a correlation factor determined by measuring a standard of pure ethanol mixed with BG11 (R^2^ > 0.99).

### Microscopy

Microscopy imaging was carried out on a Leica TCS SP5 confocal microscope. EYFP was excited at 514 nm using laser light and emission was collected between 527 and 546 nm. Autofluorescence was excited using the same wavelength and emission was collected between 605 and 710 nm. Gain and offset were identical between each sample to ensure equal treatment.

## Additional Information

**How to cite this article**: Englund, E. *et al*. Evaluation of promoters and ribosome binding sites for biotechnological applications in the unicellular cyanobacterium *Synechocystis* sp. PCC 6803. *Sci. Rep.* **6**, 36640; doi: 10.1038/srep36640 (2016).

**Publisher’s note:** Springer Nature remains neutral with regard to jurisdictional claims in published maps and institutional affiliations.

## Supplementary Material

Supplementary Information
