# Supplementary material for: Evaluation of promoters and ribosome binding sites for biotechnological applications in the unicellular cyanobacterium Synechocystis sp. PCC 6803
Source: Sci Rep. 2016 Nov 18;6:36640. doi: 10.1038/srep36640 (PMC5114575; doi:10.1038/srep36640)
Supplement: Supplementary Information [file srep36640-s1.pdf]

Supplementary material for

“Evaluation of promoters and ribosome binding sites for biotechnological applications in the unicellular cyanobacterium *Synechocystis* sp. PCC 6803".

Elias Englund, Feiyan Liang, Pia Lindberg

Department of Chemistry - Ångström, Uppsala University, Box 523, SE-751 20 Uppsala, Sweden

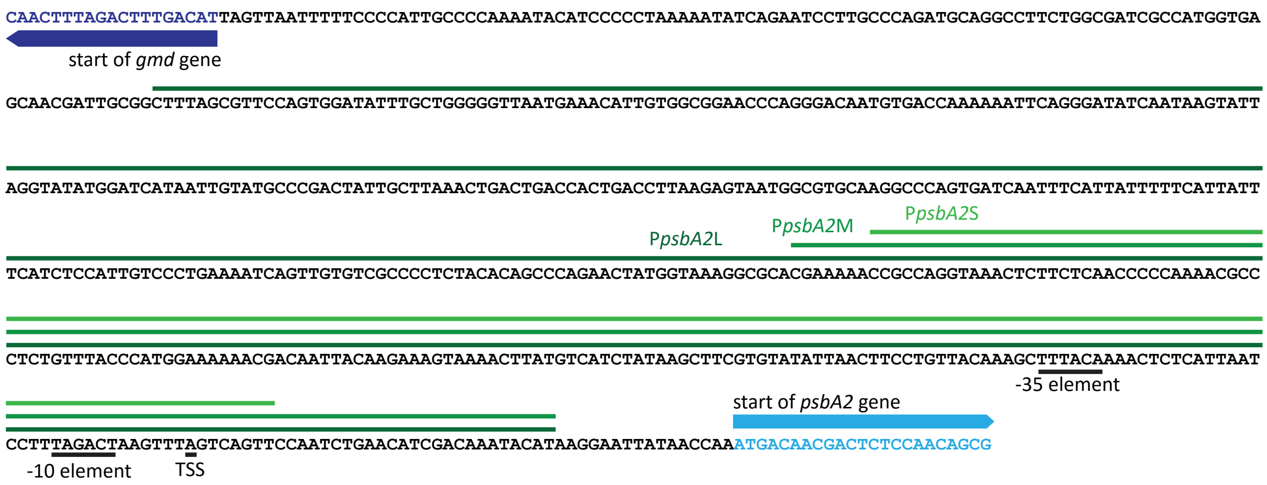

Supplementary Figure 1. Sequence of *psbA2* upstream region and the sequence used for the three P*psbA2* versions. Green lines indicate sequences used for P*psbA2*L, M and S.

Supplementary Table 1. Sequences of promoters used in the study. Transcriptional start sites (TSS) have been marked in red. For the J23xxx promoters, the TSS is 2 base pairs after the end of the promoter and for PrnpB, the TSS is 1 base pair after. We were unable to find any experimental data on PcoaT and PziaA TSS. The sequence following the promoter was ACTAGAGTAGTGGAGGTTACTAG**ATG** except for PpsbA2M and PpsbA2S which had the sequence TAGTGGAGGTTCTAGA**ATG** (RBS underlined, start codon in **bold**).

Promoter[a]	Promoter sequence
**PnrsB[1]**	TTCCACCAGCAAAATTCGCATCGCCTCTGCCTTTTTTATAACGGTCTGATCTTAGCGGGGGAAGGAGATTTTCACCTGAATTTCA<br>TACCCCCTTTGGCAGACTGGGAAAATCTTGGACAAATTCCCAATT
**PnrsD[2]**	TATTCGATTCAGTACCAAGTACTATTGCGGGGACAGGACGTTTCTCAAGGCCCTCATCAATATCCCCCCTGGGGGCATAGAATAG<br>AGATCAATTTTCTACCCCAAACCCCCACA
**PnrsS[1]**	ACCACCTCAAATTGGGAATTTGTCCAAGATTTTCCCAGTCTGCCAAAGGGGGTATGAAATTCAGGTGAAAATCTCCTTCCCCCGC<br>TAAGATCAGACCGTTATAAAAAAGGCAGAGGCG
**PcoaT**	CCCTTTAGTTTACTCAAAACCTTGACATTGACACTAATGTTAAGGTTTAGGCTGAGAAGGTAAAAATCCAAGTTAAAAAGC
**PziaA**	AGAGGTTGGCGTTAGGAGCTAGGGAAAAATTTAAACTGGATTTAGAAAATGATTTTCATCCTAACATCTTTAATATCTGAGCATA<br>TCTTCAGGTGTTTCAAGATTTGTGCTACGGTTCAAGGAGGTTTTTCTTTAAATCACGTTGGCCGCC
**PpetE[3]**	CGGCGATCGCCAAAAACAAAGAAAATTCAGCAATTACCGTGGGTAGCAAAAAATCCCCATCTAAAGTTCAGTAAATATAGCTAGA<br>ACAACCAAGCATTTTCGGCAAAGTACTATTCAGATAGAACGAGAAATGAGCTTGTTCTATCCGCCCGGGGCTGAGGCTGTATAAT<br>CTACGACGGGCTGTCAAACATTGTGATACCATGGGCAGAAGAAAGGAAAAACGTCCCTGATCGCCTTTTTGGGCACGGAGTAGGG<br>CGTTACCCCGGCCCGTTCAACCACAAGTCCCTATAGATACAATCGCCAAGAAGT
**PrbcL1A[3]**	CAGTCAATGGAGAGCATTGCCATAAGTAAAGGCATCCCCTGCGTGATAAGATTACCTTCAGAAAACAGATAGTTGCTGGGTTATC<br>GCAGATTTTTCTCGCAACCAAATAACTGTAAATAATAACTGTCTCTGGGGCGACGGTAGGCTTTATATTGCCAAATTTCGCCCGT<br>GGGAGAAAGCTAGGCTATTCAATGTTT
**PpsaA[4]**	ATCTGTGCAAGGTTTAACATCGTTATTATGAAGCGAAAACTAATTCCCTTTTTTACGCTTCCTCTATTACACTATTCTGCATAGG<br>AAACCCTTAATAGTTCATTGTCGAGCGAGGAGAACCCTGC
**PpsbA2S[5]**	CCGCCAGGTAAACTCTTCTCAACCCCCAAAACGCCCTCTGTTTACCCATGGAAAAAACGACAATTACAAGAAAGTAAAACTTATG<br>TCATCTATAAGCTTCGTGTATATTAACTTCCTGTTACAAAGCTTTACAAAACTCTCATTAATCCTTTAGACTAAGTTTAGTCAGT<br>T
**PpsbA2M[5]**	CGAAAAACCGCCAGGTAAACTCTTCTCAACCCCCAAAACGCCCTCTGTTTACCCATGGAAAAAACGACAATTACAAGAAAGTAAA<br>ACTTATGTCATCTATAAGCTTCGTGTATATTAACTTCCTGTTACAAAGCTTTACAAAACTCTCATTAATCCTTTAGACTAAGTTT<br>AGTCAGTTCCAATCTGAACATCGACAAATACAT
**PpsbA2L[5]**	CTTTAGCGTTCCAGTGGATATTTGCTGGGGGTTAATGAAACATTGTGGCGGAACCCAGGGACAATGTGACCAAAAAATTCAGGGA<br>TATCAATAAGTATTAGGTATATGGATCATAATTGTATGCCCGACTATTGCTTAAACTGACTGACCACTGACCTTAAGAGTAATGG<br>CGTGCAAGGCCCAGTGATCAATTTCATTATTTTTCATTATTTCATCTCCATTGTCCCTGAAAATCAGTTGTGTCGCCCCTCTACA<br>CAGCCCAGAACTATGGTAAAGGCGCACGAAAAACCGCCAGGTAAACTCTTCTCAACCCCCAAAACGCCCTCTGTTTACCCATGGA<br>AAAAACGACAATTACAAGAAAGTAAAACTTATGTCATCTATAAGCTTCGTGTATATTAACTTCCTGTTACAAAGCTTTACAAAAC<br>TCTCATTAATCCTTTAGACTAAGTTTAGTCAGTTCCAATCTGAACATCGACAAATACAT
**PrpnB[6]**	TTCAATGCGGTCCAATACCTCCCCTGCCCAACTGGGTAAGCTCGCGGCTCCACTGAGTAATACAGACAAGGCTAAACAGGCAAAT<br>TTTTTCATTGGTCAACTCCTAGCACCAATTTCCCAAGACTACGGAGGGGGCAATGAAGTTTCAATTAATTGGGGTCACAAACCAC<br>AGCGGCCTATGGCTCTAATCAATGGCACACTAGAAAAA
**J23101[7]**	TTTACAGCTAGCTCAGTCCTAGGTATTATGCTAGC
**J23110[7]**	TTTACGGCTAGCTCAGTCCTAGGTACAATGCTAGC
**J23119[7]**	TTGACAGCTAGCTCAGTCCTAGGTATAATGCTAGC

[a] References given for TSS

References

1 López-Maury, L., García-Domínguez, M., Florencio, F. J. & Reyes, J. C. A two-component signal transduction system involved in nickel sensing in the cyanobacterium *Synechocystis* sp. PCC 6803. *Mol. Microbiol.* **43**, 247-256, doi:10.1046/j.1365-2958.2002.02741.x (2002).

2 Foster, A. W., Patterson, C. J., Pernil, R., Hess, C. R. & Robinson, N. J. Cytosolic Ni(II) sensor in cyanobacterium: nickel detection follows nickel affinity across four families of metal sensors. *J. Biol. Chem.* **287**, 12142-12151, doi:10.1074/jbc.M111.338301 (2012).

3 Mitschke, J. *et al.* An experimentally anchored map of transcriptional start sites in the model cyanobacterium *Synechocystis* sp. PCC 6803. *Proc. Natl. Acad. Sci. U. S. A.* **108**, 2124-2129, doi:10.1073/pnas.1015154108 (2011).

4 Muramatsu, M. & Hihara, Y. Characterization of high-light-responsive promoters of the *psaAB* genes in *Synechocystis* sp. PCC 6803. *Plant Cell Physiol.* **47**, 878-890, doi:10.1093/pcp/pcj060 (2006).

5 Mohamed, A., Eriksson, J., Osiewacz, H. D. & Jansson, C. Differential expression of the *psbA* genes in the cyanobacterium *Synechocystis* 6803. *Mol. Gen. Genet.* **238**, 161-168 (1993).
6 Vioque, A. Analysis of the gene encoding the RNA subunit of ribonuclease P from cyanobacteria. *Nucleic Acids Res.* **20**, 6331-6337 (1992).
7 Davis, J. H., Rubin, A. J. & Sauer, R. T. Design, construction and characterization of a set of insulated bacterial promoters. *Nucleic Acids Res.* **39**, 1131-1141, doi:10.1093/nar/gkq810 (2011).

Supplementary Table 2. Sequences of ribosomal binding sites used in the study. The EYFP constructs were expressed by PpetE and mTagBFP constructs by PpsbA2 in this study. Following the RBS sequence were six nucleotides to the start codon. Either TACTAG**ATG** for mTagBFP constructs or TCTAGA**ATG** for EYFP constructs (start codon in **bold**). The mTagBFP constructs had a TCTAGA sequence between promoter and RBS while there was no such sequence for the EYFP constructs.

RBS	RBS sequence
**BBa_B0029**	TTCACACAGGAAACC
**BBa_B0030**	ATTAAAGAGGAGAAA
**BBa_B0031**	TCACACAGGAAACC
**BBa_B0032**	TCACACAGGAAAG
**BBa_B0033**	TCACACAGGAC
**BBa_B0034**	AAAGAGGAGAAA
**BBa_B0035**	ATTAAAGAGGAGAA
**BBa_B0064**	AAAGAGGGGAAA
***psbA2* RBS**	CATAAGGAATTAT
***rbcL* RBS**	TTATGGAGGACTG
**RBS***	TAGTGGAGGT
